# Molecular Structures and Mechanisms of Waterborne Biodegradable Polyurethane Nanoparticles

**DOI:** 10.1016/j.csbj.2018.12.007

**Published:** 2018-12-31

**Authors:** Chien-Hui Wen, Shun-Chieh Hsu, Shan-hui Hsu, Shu-Wei Chang

**Affiliations:** a Department of Civil Engineering, National Taiwan University, Taipei, Taiwan; b Inistitute of Polymer Science and Engineering, National Taiwan University, Taipei, Taiwan

**Keywords:** Molecular dynamics, Polymer, Hydrogel, Nanoparticle, biomaterial, green material

## Abstract

Biodegradable hydrogels have become promising materials for many biological applications in the past years. Recently, novel waterborne biodegradable polyurethane (WDPU) nanoparticles have been synthesized by a green water-based process, and serve as fundamental building blocks to form materials with great biocompatibility, biodegradability, and mechanical properties. However, the molecular structures and mechanisms of the WDPU nanoparticles and the relationship between the chemical compositions of the polymer segments and the material properties of the biodegradable hydrogels at macro-scale are still not well understood. In this study, we explore the fundamental mechanisms of WDPU nanoparticles through a full atomistic simulation approach to understand how the chemical compositions at the molecular level affect the molecular structures and material properties of WDPU nanoparticles. Specifically, we compare two WDPUs, i.e. PCL75LL25 and PCL75DL25, of the same hard segment composition and very similar soft segment composition [75% poly(e-caprolatone) and 25% polylactide], except the lactide in the former is L-form and in the latter is D,L-form. Our results show that the material properties of the biodegradable hydrogel can be designed by tuning the chemical compositions of the polymer segments. We find that the PCL75DL25 and PCL75LL25 have distinct molecular structures and physical crosslinks within the nanoparticles. The molecular structure of WDPU with PDLLA as soft segments is more extended, leading to more physical crosslinks between PCL segments. This study provide fundamental insights into the molecular structures and mechanisms of WDPU nanoparticles and help enabling the design of material properties of biocompatible hydrogel.

## Graphical Abstract

**Figure:**
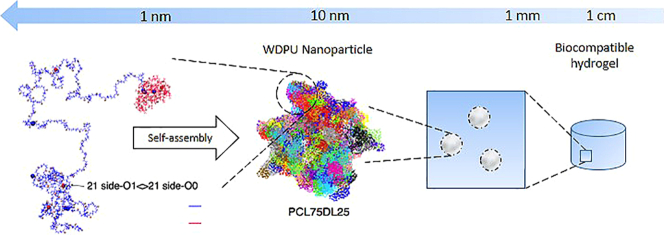

## 1 Introduction

In the past few decades, the development and applications of hydrogel have attracted increasing interest, specifically as a new class of biomaterials [[1], [2], [3], [4], [5], [6], [7], [8]]. Hydrogels, namely hydrophilic gels, are networks of polymer chains that are extensively swollen with water [1,[9], [10], [11], [12], [13], [14]]. Among hydrogels, biodegradable hydrogels are of utmost interest because they exhibit similar mechanical properties as natural soft tissues and can be degraded in an aqueous environment after their useful lifetime. Biodegradable hydrogels include natural hydrogels and synthetic type. Natural hydrogels have been gradually replaced by synthetic biodegradable hydrogels because of their strong water absorption capacity, long service life time, and a wide range of available chemical materials [10].

Recently, a novel waterborne biodegradable polyurethane (WDPU) has been synthesized and shown to have great potential in biomedical applications [[15], [16], [17], [18], [19], [20]]. It is synthesized by a green water-based process, and has great biocompatibility, biodegradability, and mechanical properties. The WDPU has been demonstrated that it can be synthesized using biodegradable polyesters as the soft segment without chemical crosslinking [18]. The properties of the WDPU nanoparticle could be adjusted by the chemical composition and ratio of the polyesters in the soft segment [15], such as PCL diol, ad PLA diol. The behavior of thermally induced self-assembly and gel formation and the degradation rate of the hydrogel can be tuned by changing the soft segments of WDPU, indicating that WDPU hydrogels with a various of material properties can be designed by changing the composition of soft segments for different biomedical applications. It has also been shown that the WDPU hydrogels are able to be applied to 3D printing for neutral stem cells (NSCs) carrier, and shown great potential in central nervous system repair [16]. The integration of biodegradable hydrogel and 3D printing technology has opened great opportunities for the design of smart biocompatible scaffolds for many applications due to the ability to access complex internal structures [[15], [16], [17], [18], [19], [20]].

WDPU nanoparticles are the fundamental building blocks of the WDPU hydrogels. The molecular structures of WDPU nanoparticles play an important role in the molecular mechanisms of sol-gel transition and the material properties of the WDPU hydrogel. However, the molecular structures and mechanisms of the WDPU and the relationship between the chemical compositions of the polymer segments and the material properties of the biodegradable hydrogels at macro-scale are still not clear. In this paper, we employed full atomistic simulations to explore the molecular structure and fundamental mechanisms of WDPU nanoparticles. WDPU nanoparticles with 75% PCL diol and 25% PLA diol soft segments (L-form or D,L-form) are simulated to investigate the relationships between the chemical composition of soft segments and the physical properties of the WDPU nanoparticles.

## 2 Materials and Methods

### 2.1 Simulation Details

#### 2.1.1 Model Construction

**Fig. 1:**
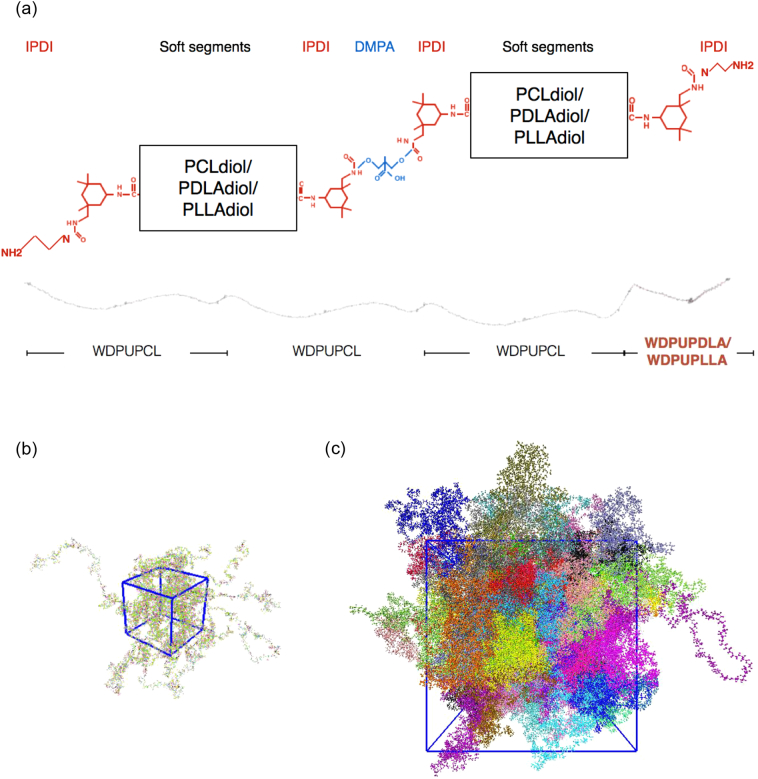
(a) Chemical composition of the polymer chains. (b) WDPU polymer chains with 75% PCL and 25% PLA are constructed. (c) Illustration of the amorphous unit cells.

Fig. 1(a) illustrates the polymer chains constructed in this work. Three different soft segments are used in this study: PCL diol, poly(d,l-lactide) diol (PDLLA diol, Mn ~ 2000 Da) and poly(l-lactide) diol (PLLA diol, Mn ~ 2000). The WDPU with PCL diol as both soft segments is named WDPUPCL. The WDPU with PDLLA diol as both soft segments is named WDPUPDLA. The WDPU with PLLAdiol as both soft segments is named WDPUPLLA. Each WDPU polymer chain is consisted of 4 repeating units with two soft segments. PCL and PDLLA are mixed in 3:1 M ratio to construct the PCL75DL25 nanoparticle while PCL and PLLA are mixed in 3:1 M ratio to construct the PCL75LL25 nanoparticle (Fig. 1(a)).

We use Materials Studio to construct WDPU monomers according to the previously published synthesis procedure [15]. Each chain contains 3070 atoms and the molecular weight is about 39,018 Da. We use Amorphous Cell to construct the initial amorphous cells for PCL75DL25 and PCL75LL25 as shown in Fig. 1(b). Each initial unit cell consists 10 chains of polymers. To equilibrate the amorphous structure, we firstly run NPT simulation at 500 K for 0.6 ns to speed up the process, then cool down the temperature to 300 K for 0.2 ns to reach equilibrium at the room temperature. The equilibrated unit cell is then replicated in all three directions to construct a larger amorphous packing nanoparticle (Fig. 1(c)) to simulate the self-assembly of the nanoparticle in a larger box in vacuum. The final model consists of 245,600 atoms.

#### 2.1.2 Force Field

We use consistent valence force field (CVFF) [21] to simulate the molecular structure of the WDPU nanoparticles. The CVFF originally applied to biological system [22] and is a prototype of the consistent force field (CFF) and its later derivatives (the polymer consistent force field PCFF and COMPASS). Terms in CVFF included a bond stretching or compression, an angle bending, torsion angle twisting and out-of-plane deformation of a planar system, five cross-coupling terms between bond deformations, angle bending, a bond deformation and an angle bending, a torsion angle twisting and the two associated angle bending, and coupling between out-of-plane deformations, and repulsive, dispersive, and Coulombic interactions between nonbonded atoms [23]. The energy description is shown in Eq. (1). The first four terms in Eq. (1) included a Morse potential for bond stretching and compression (b), a harmonic term for angle bending (θ), cosine torsional (ϕ) and out-of-plane torsional terms (χ). *D*_*b*_, *H*_*θ*_, *H*_ϕ_, and *H*_χ_ are the force constants for the corresponding intramolecular deformations. *b*_0_ and *θ*_0_ are the equilibrium bond length and angle. The next five terms are cross terms that account for interactions between the four types of internal coordinates. *F*_*ij*_ are the force constants for the cross terms. The last two terms represents Lennard-Jones and Coulombic terms for nonbonded interaction. ε and *r*^∗^ are the parameters for the nonbonded repulsive and dispersive interactions, and *q*_*i*_ are the partial charges on each atom [[23], [24], [25]].

(1) $$E_{\mathrm{pot}}=\sum_{b}D_{b}\left[1-e^{-\alpha \left(b-b_{0}\right)}\right]+\sum_{\theta }H_{\theta }{\left(\theta -\theta_{0}\right)}^{2}+\sum_{\phi }H_{\phi }\left[1+s\cos\left(\mathrm{n\phi}\right)\right]+\sum_{\chi }H_{\chi }\chi^{2}+\sum_{b}\sum_{b^{\prime }}F_{bb^{\prime }}\left(b-b_{0}\right)\left(b^{\prime }-b_{0}^{\prime }\right)+\sum_{\theta }\sum_{\theta^{\prime }}F_{\theta \theta^{\prime }}\left(\theta -\theta_{0}\right)\left(\theta^{\prime }-\theta_{0}^{\prime }\right)+\sum_{b}\sum_{\theta }F_{\mathrm{b\theta}}\left(b-b_{0}\right)\left(\theta -\theta_{0}\right)+\sum_{\phi }F_{\phi \theta \theta^{\prime }}\cos\phi \left(\theta -\theta_{0}\right)\left(\theta^{\prime }-\theta_{0}^{\prime }\right)+\sum_{\chi }\sum_{\chi^{\prime }}F_{\chi \chi^{\prime }}\chi \chi^{\prime }+\sum \varepsilon \left[{\left(\frac{r^{*}}{r}\right)}^{12}-2{\left(\frac{r^{*}}{r}\right)}^{6}\right]+\sum \frac{q_{i}q_{j}}{\varepsilon r_{\mathrm{ij}}}$$

### 2.2 Analysis of Molecular Structures

#### 2.2.1 Radius of Gyration

Radius of gyration, R_g_, is a useful representation to measure and characterize the size of complex polymer configurations. We compute the average of the radius of gyration of the last 0.1 ns after the simulations of 2 ns. We use Eq. (2) to calculate the radius of gyration of WDPU nanoparticles. In Eq. (2), $\vec{r_{i}}$ is the position of the *i*-th atom and $\vec{r_{\mathrm{cm}}}$ is the center of mass of the WDPU nanoparticle.

(2) $$R_{g}=\sqrt{\frac{1}{N}\sum_{i=1}^{N}{\left(\left|\vec{r_{i}}-\vec{r_{\mathrm{cm}}}\right|\right)}^{2}}$$

#### 2.2.2 Eccentricity

To understand if the differences on the radius of gyration are resulted from the shape of nanoparticles, we calculate the eccentricity (ρ_eccen_) of the nanoparticles to investigate the structures of nanoparticles. The eccentricity measures the symmetry of WDPU nanoparticles. If the value is close to zero, it implies a high symmetry, while if the value is larger, it implies less symmetry. We use Eq. (3) to calculate the eccentricity of WDPU nanoparticles [26]. In Eq. (3), *I*_*min*_ is the minimum value of the tri-axial moment of inertia and *I*_*avg*_ is the average value of the tri-axial moment of inertia. We compute the average of the eccentricity of the last 0.1 ns after the simulations of 2 ns.

(3) $$P_{\mathrm{eccen}}=1-\frac{I_{\min}}{I_{\mathrm{average}}}$$

#### 2.2.3 Surface Area

Solvent-accessible surface area of atoms in the nanoparticle is calculated by using the assigned radius for each atom with an extended radius for 1.4 Å to find the points on a sphere that are exposed to solvent. To normalize the surface area, we calculate the surface area of WDPU nanoparticles (A_m_), and divided by surface area when the nanoparticle is spherical (A_s_). Values close to one imply a higher spherical symmetry while a larger value indicates that the nanoparticle has unsmooth surface or irregular shape. We compute the average of the surface area of the last 0.1 ns after the simulations of 2 ns.

#### 2.2.4 End to End Distance

End to end distance is a parameter to describe the conformation of polymer chains. To calculate the end to end distance of polymer chains, we define the atomic positions of the Nitrogen atoms on N-C-C-N as the ends of a segment, and the distance between these two atomic positions are considered as the end to end distance of segments.

## 3 Results

### 3.1 Nanoparticle Characterization

**Fig. 2:**
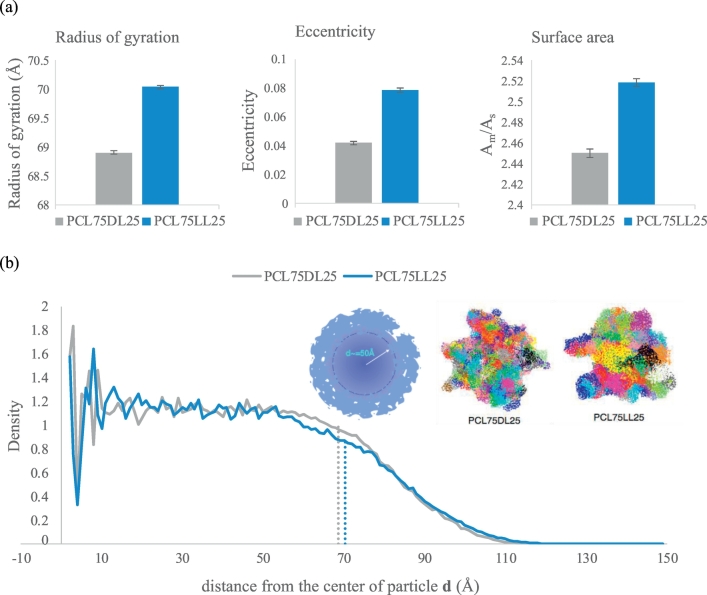
Nanoparticle characterizations. (a) Radius of gyration, eccentricity and surface area of WDPN nanoparticles. The error bars are the standard deviations from the ensemble average of the last 0.1 ns simulations. (b) Analysis of the density distribution of WDPU nanoparticles. The results show that the PCL75LL25 nanoparticle has a more irregular shape. There is no differences on the core density between the PCL75DL25 and PCL75LL25 nanoparticles but there is significant differences on the surface morphology when the PLLA soft segment is replaced with PDLLA soft segments. The molecular packing on the surface of nanoparticle is less dense for the PCL75DL25 nanoparticle.

To characterize the structures of WDPU nanoparticles, we analyze the radius of gyrations, eccentricity, and surface area of PCL75DL25 and PCL75LL25 nanoparticles. Fig. 2 (a) shows the results. We find that the PCL75LL25 nanoparticle has a radius of gyration with a value of 70.01 ± 0.03 Å, while the PCL75DL25 nanoparticle has a slightly smaller radius of gyration of 68.90 ± 0.03 Å. The results of the eccentricity show that the PCL75DL25 nanoparticle has a value of **ρ**_eccen_ = 0.039 **±** 0.004 and the value of the PCL75LL25 nanoparticle is 0.079 ± 0.004, suggesting that the shape of the PCL75LL25 nanoparticle is more irregular. For the surface area, there is no significant differences on the surface area between the PCL75DL25 and the PCL75LL25 nanoparticles. It is worth noting that these results show that both PCL75DL25 and PCL75LL25 have unsmoothed surface and the PCL75LL25 nanoparticle has a more irregular and unsymmetrical shape.

Since both polymers have the same molecular weights, the differences on the radius of gyration might be resulted from either the change on the packing of molecules within the nanoparticles or the change on the surface morphology. We analyze the density distributions of both nanoparticles as shown in Fig. 2 (b). In Fig. 2 (b), we find that both nanoparticles have the same core density when the radius is smaller than 50 Å. These results indicate that there is no difference on the density in the center between different WDPU nanoparticles, while there is significant differences on the surface morphology between different WDPU nanoparticles. From the distance of 55 Å, the density of both PCL75DL25 and PCL75LL25 nanoparticles start to decrease indicating that the packing of polymer chains on the surfaces of both nanoparticles are much looser than the molecular packing in the center of the nanoparticles. Although both nanoparticles have a radius of gyration ~70 Å, the density of PCL75DL25 and PCL75LL25 decrease slowly and goes to 0 when the radius exceeds 110 Å. It is worth noting that from 50 to 80 Å, the PCL75DL25 has a larger density while a reverse trend is found for the distance from 80 to 120 Å. The density of PCL75DL25 reaches 0 at a distance smaller than the PCL75LL25. These results suggest that the self-assembly of PCL75LL25 nanoparticle is more loosely on the surface when compare with the PCL75DL25 nanoparticle. That is, the slightly larger radius gyration of the PCL75LL25 is not resulted from different packing density in the center of the nanoparticle but resulted from the differences on the surface morphology of the WDPU nanoparticles. We anticipate that the loose surface packing on both nanoparticles play important roles in the sol-gel transition. The loose surface packing allows polymer chains on the surface to move more freely and interact with other nanoparticles in solutions at higher temperature to initiate the physical crosslinks between nanoparticles and eventually form networks and further initiate the hydrogel formation. The larger radius of gyration and the looser packing on the surface of the PCL75LL25 nanoparticle provide molecular insights into its faster sol-gel transition time observed in experimental results.

### 3.2 Molecular Structure of WDPU Nanoparticles

**Fig. 3:**
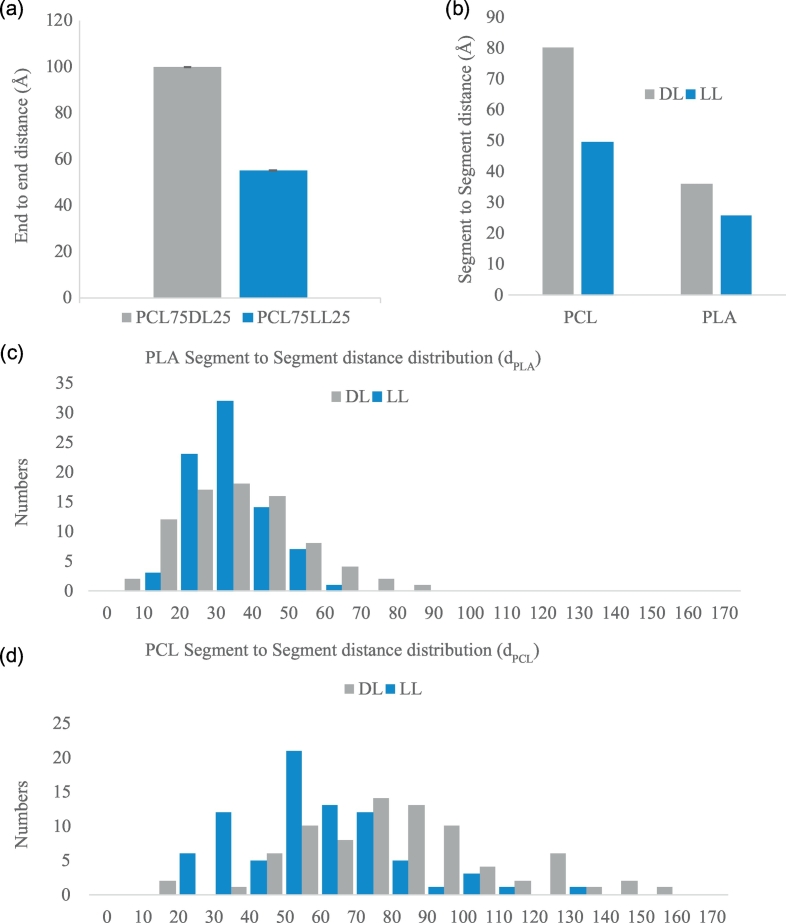
Analysis of the end to end distance of polymer chains in WDPU nanoparticles. (a) The end to end distances of polymer chains in WDPU nanoparticles. (b) PCL and PLA segment to segment distances in WDPU nanoparticles. The error bars are the standard deviations from the ensemble average of the last 0.1 ns simulations. (c) the distribution of the end to end distances of PLA segments in WDPU nanoparticles. (d) the distribution of the end to end distances of PCL segments in WDPU nanoparticles.

The characterizations of WDPU nanoparticles show that the PCL75LL25 nanoparticle has smaller density in the surface layer and higher value of eccentricity. The packing and self-assembly of the nanoparticles are determined by the differences on the properties of each chain and the interactions between polymer chains. To understand the molecular structural differences between the PCL75DL25 and PCL75LL25 nanoparticles, we further analyze the end to end distances of polymer chains as shown in Fig. 3 (a). We find that although there is not much difference on the characterization of the shape between the PCL75DL25 and PCL75LL25 nanoparticles, there are significant differences at the molecular level between the PCL75DL25 and PCL75LL25 nanoparticles. The end to end distances of the polymer chains in the PCL75DL25 nanoparticles (99.976 ± 0.134 Å) is almost two times larger than the end to end distance of the polymer chains in the PCL75LL25 nanoparticles (54.990 ± 0.228 Å). It is worth noting that all the polymer chains in both WDPU nanoparticles have similar contour length, indicating that the polymer chains in the PCL75DL25 nanoparticle is much stiffer than the polymer chains in the PCL75LL25 nanoparticle. These results suggest that PLLA soft segments significantly cause shorter end to end distances of polymer chains while the PDLLA soft segments do not have the same effects.

Since all the polymer chains in both nanoparticles have 75% PCL segments and 25% PLA segments (PDLLA soft segments in PCL75DL25 and PLLA soft segments in PCL75LL25), we further analyze the segment to segment distances of PCL segments and PLA segments in both nanoparticles as shown in Fig. 3 (b). The distributions of the segment to segment distances are shown in Fig. 3 (c) and (d). The results reveal that there are significant differences on the segment to segment distances of the PCL soft segments. The PLLA soft segments significantly cause shorter segment to segment distances of PCL soft segments in the PCL75LL25 nanoparticle while the PCL soft segments have much larger segment to segment distances in the PCL75DL25 nanoparticle. These results suggest that PDLLA increases the stiffness of the PCL soft segments in the PCL75DL25 nanoparticle. The shorter end to end distances, i.e. lower stiffness, for the PCL75LL25 nanoparticle suggest that when solvating in the solution, the polymer chains are easier to reach out and form physical crosslinks with other nanoparticles.

**Fig. 4:**
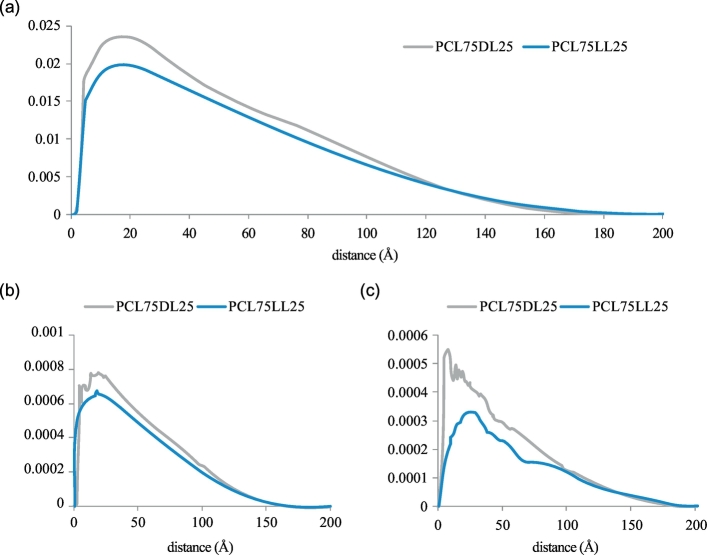
Analysis of the radial distribution function between different polymer chains. (a) Radial distribution of the polymer chains. (b) Radial distribution of the PCL segments. (c) Radial distribution of the PLA segments.

We further analyze the radial distribution function (RDF) between different polymer chains to study the packing behaviors of the PCL75DL25 and PCL75LL25 nanoparticles. Fig. 4 (a) shows the RDF between the polymer chains. The peak of the RDF occurs at 17.75 Å for the PCL75DL25 nanoparticle while at 16.75 Å for the PCL75LL25 nanoparticle, suggesting the packing of both nanoparticles are similar from the point of view of the polymer chains. It is worth noting that the RDF of the PCL75DL25 nanoparticle drops to zero at a shorter distance when compared with the RDF of the PCL75LL25 nanoparticle. These results are consistent with the analysis of the density distribution of the nanoparticles as shown in Fig. 2 (b). Although there is no significant difference on the RDF of the polymer chains, we find that there is significant difference if we analyze the RDF of the PCL and PLA soft segments separately for the nanoparticles. Fig. 4 (b) and (c) show the RDF of the PCL soft segments and PLA soft segments. The RDF of the PCL soft segments are similar for both nanoparticles while there is a significant difference on the RDF of the PLA soft segments as shown in Fig. 4 (c). The first peak of the RDF of the PLA soft segments occurs at 8.25 Å for the PCL75DL25 nanoparticle while at 23.75 Å for the PCL75LL25 nanoparticle. Together with the results from the analysis of the end to end distances and segment to segment distances as shown in Fig. 3, our results reveal that the PCL and PLA soft segments are more extended in the PCL75DL25 nanoparticle resulted in a packing of polymer chains with shorter distances between PLA soft segments in the PCL75DL25 nanoparticle.

### 3.3 Intermolecular and Intramolecular Hydrogen Bonds in WDPU Nanoparticles

**Fig. 5:**
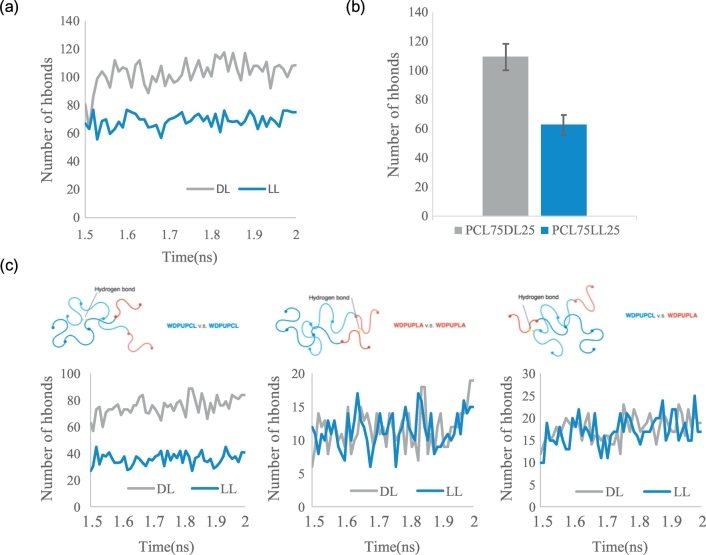
Analysis of the number of hydrogen bonds in the PCL75DL25 and PCL75LL25 nanoparticles. The hydrogen bond is defined when the distance between the donor and acceptor is less than 3.0 Å and the angle is less than 30 degrees. (a) The number of total hydrogen bonds versus simulation time. (b) The average number of total hydrogen bonds. The error bars are the standard deviations from the ensemble average of the last 0.1 ns simulations. (c) Analysis of the number of three different kinds of hydrogen bonds.

The analysis of hydrogen bonds in the WDPU nanoparticles are shown in Fig. 5 to investigate the differences on the molecular interactions between the PCL75DL25 and PCL75LL25 nanoparticles. We compute the average of the hydrogen bonds of the last 0.1 ns after the simulations of 2 ns, with cutoff 30 Å and angle 30. The number of hydrogen bonds in the PCL75DL25 nanoparticle is 108 ± 6.48 while the number of hydrogen bonds in the PCL75LL25 nanoparticle is 67.909 ± 6.685. That is, there are about 50% more physical crosslinks in the PCL75DL25 nanoparticle. To reveal the molecular origin of the significant difference on the number of hydrogen bonds in PCL75DL25 and PCL75LL25 nanoparticles, we further analyze the hydrogen bonds of PCL75DL25 and PCL75LL25 by categorizing the type of hydrogen bonds into three groups. We named the segment with PCL as soft segments WDPUPCL, and the segment with PLA as soft segment WDPUPLA. Thus the three categories are: (1) hydrogen bonds between WDPUPCL and WDPUPCL; (2) hydrogen bonds between WDPUPLA and WDPUPLA; (3) hydrogen bonds between WDPUPCL and WDPUPLA. The results are shown in Fig. 5 (c). Surprisingly, our results reveal that the major difference does not occur between WDPUPLA and WDPUPLA. The major difference occurs between WDPUPCL instead, although both nanoparticles have the same WDPUPCL soft segments. These results suggest that the PDLLA soft segment increases the number of hydrogen bonds between the PCL soft segments in the PCL75DL25 nanoparticle. Our findings provide a molecular evidence that the PLLA soft segments induces the crystalline of PCL soft segments as observed in previous experimental studies [27].

**Fig. 6:**
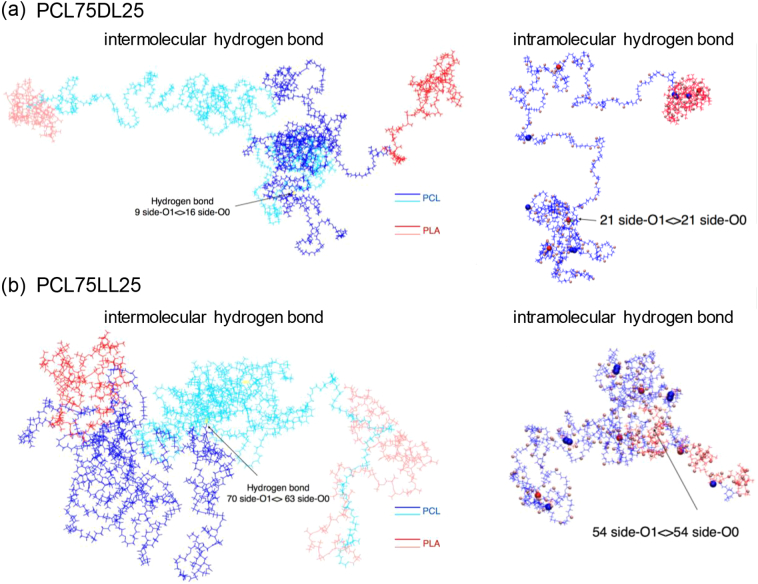
Illustration of the intermolecular and intramolecular hydrogen bond conformations in WDPU nanoparticles. (a) PCL75DL25 and (b) PCL75LL25.

We anticipate that the difference in the location and number of hydrogen bond affects the configurations of the WDPU nanoparticles. From previous analysis, we know that the major difference of hydrogen bond number is between WDPUPCL segments, suggesting that the PLA soft segment enhances the interactions between PCL segments in the nanoparticles. Further analysis of the simulation results shows that the conformation of PCL75DL25 and PCL75LL25 are different. Fig. 6 illustrates the interactions between polymer chains of PCL75DL25 and PCL75LL25, the color pink and red represent WDPUPLA, and WDPUPCL is in blue and cyan. For PCL75DL25, WDPUPLA have larger end to end distances and tend to tangle together resulting a separation of the PCL soft segments, while for PCL75LL25, PLA segments and PCL segments prefer to assemble together resulting shorter end to end distances. Fig. 6 also illustrates the intramolecular hydrogen bonds of PCL75DL25 and PCL75LL25 nanoparticles. These results show that the optical rotation of PLA would significantly affect the conformation of the whole WDPU and the number of physical crosslinks within the nanoparticle. PDLLA would result a configuration in which molecules are more extended, while the configuration of WDPU with PLLA as soft segment is more staggered.

## 4 Discussion and Conclusions

Full atomistic simulations of the PCL75DL25 and PCL75LL25 nanoparticles reveal that both nanoparticles do not have perfect spherical shapes. The PCL75LL25 nanoparticle is more irregular and asymmetry. These results are consistent with the experiment results that the size of PCL80LL20 nanoparticle is larger than the size of PCL80DL20 nanoparticle, and the structure of PCL80LL20 particle is relatively loose [16]. The analysis of the density distribution of the nanoparticle show that there is no difference on the center of the nanoparticles. Both nanoparticles have loose packing on the surface allowing them to interact with other nanoparticles in the solution to form hydrogels at high solid content and high temperature. We find that the PCL75LL25 has a larger surface layer and lower packing density in the surface layer. This may be the molecular origin of the faster sol-gel transition for the PCL75LL25.

In order to understand the molecular structures of the WDPU nanoparticles, end to end distance of each polymer chains and hydrogen bonds are analyzed. We find that there are significant differences on the end to end distance and number of hydrogen bonds between the PCL75DL25 and PCL75LL25 nanoparticles. The polymer chains in the PCL75DL25 has larger end to end distances. The structure of WDPU with PDLLA as soft segments is more extended, while the distance between PLLA and PCL is close, which may reduce the occurrence of hydrogen bonding in PCL. There are more hydrogen bonds between PCL segments in the PCL75DL25 nanoparticle. Our results reveal that the optical rotation of PLA segments would significantly affect the molecular structures of PCL segments and the number of physical crosslinks between PCL segments. These results provide a possible molecular mechanism on how the WDPU nanoparticles form hydrogels and reveal the molecular origin of the faster sol-gel transition for the PCL75LL25. Our findings suggest that adding a small amount of PDLLA soft segment could significantly increase the number of hydrogen bonds within the nanoparticles and increases the sol-gel transition behavior. The fundamental insights into the molecular structure and mechanism of WDPU nanoparticles could help enabling the design of material properties of biocompatible hydrogel by precisely tuning the chemical compositions in the soft segments of the WDPU.

## Conflicts of Interest

The authors declare no conflict of interest.
